# Arrhythmogenic calmodulin variants D131E and Q135P disrupt interaction with the L‐type voltage‐gated Ca^2+^ channel (Ca_v_1.2) and reduce Ca^2+^‐dependent inactivation

**DOI:** 10.1111/apha.14276

**Published:** 2025-01-17

**Authors:** Nitika Gupta, Ella M. B. Richards, Vanessa S. Morris, Rachael Morris, Kirsty Wadmore, Marie Held, Liam McCormick, Ohm Prakash, Caroline Dart, Nordine Helassa

**Affiliations:** ^1^ Department of Biochemistry, Cell and Systems Biology, Institute of Systems, Molecular and Integrative Biology, Faculty of Health and Life Sciences University of Liverpool Liverpool UK

**Keywords:** calmodulin, cardiac arrhythmia, catecholaminergic polymorphic ventricular tachycardia, Ca_v_1.2, CPVT, long QT syndrome, LQTS, L‐type voltage‐gated Ca^2+^ channel

## Abstract

**Aim:**

Long QT syndrome (LQTS) and catecholaminergic polymorphism ventricular tachycardia (CPVT) are inherited cardiac disorders often caused by mutations in ion channels. These arrhythmia syndromes have recently been associated with calmodulin (CaM) variants. Here, we investigate the impact of the arrhythmogenic variants D131E and Q135P on CaM's structure–function relationship. Our study focuses on the L‐type calcium channel Ca_v_1.2, a crucial component of the ventricular action potential and excitation–contraction coupling.

**Methods:**

We used circular dichroism (CD), ^1^H‐^15^N HSQC NMR, and trypsin digestion to determine the structural and stability properties of CaM variants. The affinity of CaM for Ca^2+^ and interaction of Ca^2+^/CaM with Ca_v_1.2 (IQ and NSCaTE domains) were investigated using intrinsic tyrosine fluorescence and isothermal titration calorimetry (ITC), respectively. The effect of CaM variants of Ca_v_1.2 activity was determined using HEK293‐Ca_v_1.2 cells (B'SYS) and whole‐cell patch‐clamp electrophysiology.

**Results:**

Using a combination of protein biophysics and structural biology, we show that the disease‐associated mutations D131E and Q135P mutations alter apo/CaM structure and stability. In the Ca^2+^‐bound state, D131E and Q135P exhibited reduced Ca^2+^ binding affinity, significant structural changes, and altered interaction with Ca_v_1.2 domains (increased affinity for Ca_v_1.2‐IQ and decreased affinity for Ca_v_1.2‐NSCaTE). We show that the mutations dramatically impair Ca^2+^‐dependent inactivation (CDI) of Ca_v_1.2, which would contribute to abnormal Ca^2+^ influx, leading to disrupted Ca^2+^ handling, characteristic of cardiac arrhythmia syndromes.

**Conclusions:**

These findings provide insights into the molecular mechanisms behind arrhythmia caused by calmodulin mutations, contributing to our understanding of cardiac syndromes at a molecular and cellular level.

## 1 INTRODUCTION

Long QT syndrome (LQTS) and catecholaminergic polymorphic ventricular tachycardia (CPVT) are inherited cardiac ventricular arrhythmic disorders with an overall estimated prevalence of approximately 1 in 2000 and 1 in 10 000, respectively.^1^, ^2^ LQTS is characterized by prolonged QT intervals on an electrocardiogram (ECG), indicative of longer ventricular depolarization.^1^, ^3^, ^4^, ^5^ CPVT patients often show a normal ECG at rest, but present a polymorphic ventricular tachycardia phenotype in response to emotional stress or exercise.^6^, ^7^ If left untreated, these cardiac arrhythmia syndromes can deteriorate into an oscillatory pattern known as “torsades de pointes,” leading to syncope and even cardiac arrest. Mutations in the ion channels K_v_7.1, hERG, CASQ2, and RyR2 account for over 70% of cases; however, genetic screening of patients has revealed an association between LQTS/CPVT and genetic variation in calmodulin (CaM).^8^, ^9^, ^10^, ^11^, ^12^, ^13^, ^14^, ^15^, ^16^, ^17^, ^18^, ^19^, ^20^, ^21^, ^22^, ^23^

CaM is a ubiquitous, highly conserved, 148 amino acid long calcium (Ca^2+^) sensing protein encoded for by three genes, *CALM1‐3*. All three genes encode for an identical protein, this genetic redundancy indicates the importance of functional CaM in physiology. CaM interacts with Ca^2+^ ions via EF‐hand motifs, which comprise a helix–loop–helix motif. CaM contains four EF‐hands, with two present in each lobe of the protein. The amino acid sequences of these motifs are highly conserved between EF‐hands 1–4.^24^ The C‐lobe of CaM displays a relatively high affinity for Ca^2+^ (*K*_d_ ~ 1 μM), while the N‐lobe's Ca^2+^ affinity is ~10 times lower.^25^, ^26^, ^27^ This allows CaM to detect a range of cytosolic Ca^2+^ concentrations ([Ca^2+^]_cyt_).^28^ In the absence of Ca^2+^ (apo form), CaM adopts a “closed” conformation with high degrees of flexibility.^29^ Upon Ca^2+^ binding, CaM undergoes structural changes, leading to an “open” and more stable, elongated conformation often likened to a dumbbell shape (Ca^2+^/CaM).^30^ This exposes hydrophobic pockets in the N‐ and C‐lobes allowing for interaction with CaM binding domains (CaMBDs) on other proteins.^25^ The flexibility of the linker region helps to maintain the ability of CaM to wrap around and interact with a broad range of targets, including the L‐type voltage‐gated Ca^2+^ channel Ca_v_1.2.^31^

During the ventricular action potential, activation of Ca_v_1.2 leads to an influx of Ca^2+^ ions into the myocyte, creating a microdomain of high [Ca^2+^] in the region of the dyadic cleft.^32^ This localized increase in intracellular [Ca^2+^] activates the cardiac ryanodine receptor (RyR2) and promotes Ca^2+^ efflux from the sarcoplasmic reticulum (SR) into the cytosol, in a process known as Ca^2+^‐induced Ca^2+^ release (CICR). The cytosolic Ca^2+^ ions are then able to interact with the contractile machinery of the myocyte, leading to cardiac muscle contraction. [Ca^2+^]_cyt_ is then cleared by the SR Ca^2+^‐ATPase (SERCA), the plasma membrane Ca^2+^‐ATPase (PMCA), and the Na^+^ Ca^2+^ exchanger (NCX). This coupled with the closure of RyR2 and Ca_v_1.2 channels, leads to termination of the Ca^2+^ signal and muscle relaxation.^31^, ^33^ Regulation of Ca_v_1.2 activity therefore plays a crucial role in excitation–contraction coupling in the heart.

It has been shown that CaM binds to and regulates the activity of Ca_v_1.2.^27^ In Ca_v_1.2, several CaMBDs have been identified including the IQ‐like domain (IQ) and the N‐terminal spatial Ca^2+^ transforming element (NSCaTE). Both domains have been implicated in Ca^2+^‐dependent inactivation (CDI) of the channel.^34^, ^35^, ^36^, ^37^, ^38^ At resting Ca^2+^ concentrations, apo/CaM pre‐associates with the IQ domain,^39^, ^40^, ^41^, ^42^ which enables rapid transduction of Ca^2+^ signals to the channel during activation. When [Ca^2+^]_cyt_ rises, Ca^2+^/CaM binds to NSCaTE,^36^, ^37^, ^38^, ^43^ allowing the formation of a bridge between the C‐ and N‐termini of Ca_v_1.2 and causing CDI, terminating the inward Ca^2+^ current. CDI is a critical regulatory process that tunes the kinetics of Ca^2+^ entry. It is a key physiological process to limit excess Ca^2+^ influx and disruption of this feedback mechanism in the cardiac myocytes may lead to lethal cardiac arrhythmias.

**FIGURE 1:**
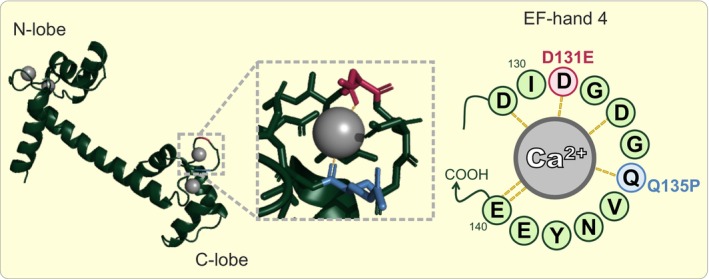
Representation of Ca^2+^/CaM highlighting arrhythmia‐associated mutations. Cartoon representation of Ca^2+^‐CaM (PDB 1CLL) showing the positions of the D131E (red) and Q135P (blue) mutations in the Ca^2+^‐coordinating positions of the EF‐hand. Dashed gray lines indicate the interaction of residues with Ca^2+^.

CaM variants D131E and Q135P have been clinically associated with LQTS/CPVT, with patients presenting severe arrhythmia phenotypes at a young age.^14^ These single‐point missense mutations are located in the C‐lobe of CaM, in Ca^2+^ coordinating residues in EF‐hand 4 (Figure 1). D131E and Q135P show altered Ca^2+^ binding properties, with Q135P adopting a different conformation when bound to the IQ domain of Ca_v_1.2.^14^, ^44^ However, little is known about the interaction of these disease variants with other Ca_v_1.2 domains, or the functional consequences on Ca_v_1.2 currents and CDI. In this article, we used a multidisciplinary approach combining protein biophysics, structural biology, and electrophysiology to determine the structure–function relationship of disease‐associated CaM variants and Ca_v_1.2. We demonstrate that arrhythmogenic CaM variants D131E and Q135P dramatically reduce CDI of Ca_v_1.2, most likely through altered CaM structure and impaired interaction with Ca_v_1.2‐IQ and Ca_v_1.2‐NSCaTE domains. These findings provide an insight into the molecular basis for cardiac arrhythmias caused by CaM mutations.

## 2 RESULTS

### 2.1 Arrhythmia‐associated mutations cause subtle changes in apo/CaM structure and stability

#### 2.1.1 Structural analysis of apo/CaM using circular dichroism (CD) and nuclear magnetic resonance (NMR)

**FIGURE 2:**
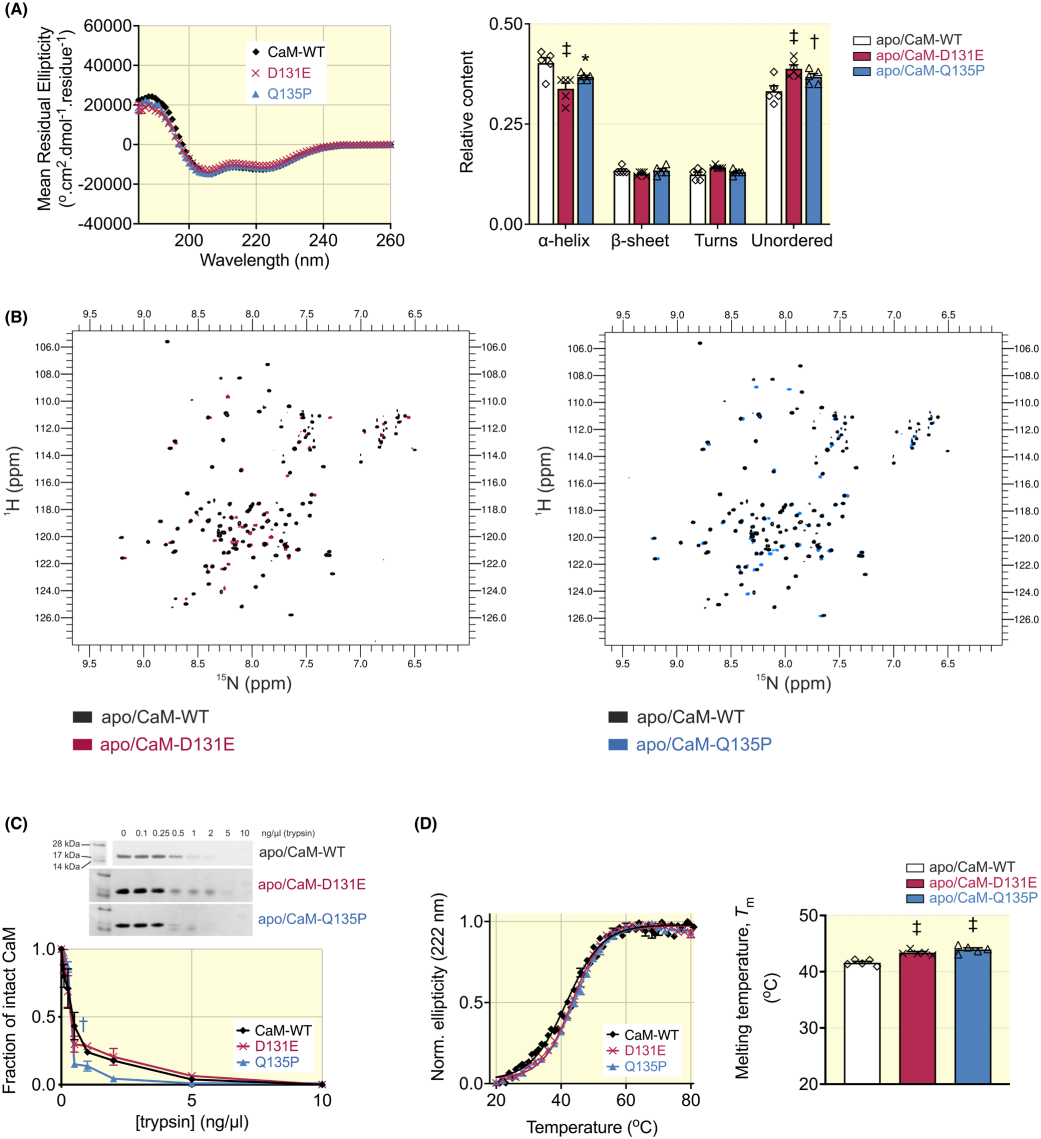
Arrhythmia‐associated mutations D131E and Q135P induce subtle changes in apo/CaM structure and stability. (A) Average far‐UV CD spectra of apo/CaM isoforms and relative secondary structure content estimations based on the CDSSTR prediction algorithm (reference data set 7). Data are presented as mean ± SEM (CaM‐WT, *n* = 5; D131E, *n* = 5; Q135P, *n* = 5) and differences between groups were determined using a two‐way ANOVA with Dunnett's multiple comparisons tests (*p < 0.05, ^†^ *p* < 0.01, ^‡^ *p* < 0.001). (B) Overlays of ^1^H‐^15^N HSQC NMR spectra of apo/CaM‐WT with variants D131E or Q135P. Each peak in the spectra represents an amide bond, with its position determined by its chemical shift in the H and N dimensions (x and y axes, respectively). (C) Sensitivity of apo/CaM variants to trypsin degradation using SDS‐PAGE (Coomassie staining). The band at 16.8 kDa representing intact CaM was quantified by densitometry and normalized. Data are presented as mean ± SEM (CaM‐WT, *n* = 3; D131E, *n* = 5; Q135P, *n* = 3) and differences between groups were determined using a one‐way ANOVA with Dunnett's multiple comparisons tests (^†^ *p* < 0.01). (D) Thermal stability of apo/CaM measured by CD spectroscopy. Normalized change in ellipticity at 222 nm of CaM isoforms as a function of temperature, was fitted to the Boltzmann equation to calculate melting points (*T*_m_). Data are presented as mean ± SEM (CaM‐WT, *n* = 5; D131E, *n* = 5; Q135P, *n* = 5) and differences between groups were determined using a one‐way ANOVA with Dunnett's multiple comparisons tests (^‡^ *p* < 0.001).

To determine the effect of arrhythmia‐associated mutations on the structure and stability of apo/CaM, we used a combination of CD, ^1^H‐^15^N HSQC NMR, and biochemical assays. Secondary structure content was obtained from CD far‐UV spectra (Figure 2A). Apo/CaM‐WT was composed of 40 ± 1% *α*‐helices, 13 ± 1% *β*‐sheets, 12 ± 1% turns, and 33 ± 1% unordered. For both D131E and Q135P, we observed a significant reduction in the *α*‐helical content (34 ± 1% for D131E, 37 ± 1% for Q135P) and an increase in unordered structures (39 ± 1% for D131E, 37 ± 1% for Q135P). Further structural characterization of apo/CaM variants was achieved through ^1^H‐^15^N HSQC NMR spectroscopy, which provides information on the tertiary structure of CaM based on the chemical environment of the residues in the protein. Spectral overlays between apo/CaM‐WT and the disease‐associated variants demonstrated high spectral conformity with many peaks overlapping, suggesting that mutations only subtly affect apo/CaM structure (Figure 2B).

#### 2.1.2 Enzymatic and thermal stability of apo/CaM variants

Protein vulnerability to proteolytic degradation is dependent on the accessibility of protease cleavage sites, therefore changes in proteolytic stability may reflect alterations to CaM conformation. The susceptibility of apo/CaM variants to digestion by trypsin was investigated to gain insight into the effect of the mutations on the overall structure of the protein (Figure 2C). For all CaM variants, almost complete digestion was achieved with 5 ng/μL [trypsin], leaving only a small, undigested fraction (0.04 ± 0.01) of intact CaM. Apo/CaM‐Q135P showed an increased susceptibility to trypsin digestion at 0.5 ng/μL with a remaining fraction of intact apo/CaM‐Q135P of 0.15 ± 0.02, compared with 0.43 ± 0.10 for apo/CaM‐WT. Apo/CaM stability was further explored by thermal denaturation, where protein unfolding was measured by a reduction in α‐helical content using CD spectroscopy (Figure 2D). Subtle but significant differences in melting temperature (*T*_m_) were observed for the arrhythmia‐associated CaM variants D131E and Q135P. *T*_m_ for apo/CaM‐WT was increased from 41.6 ± 0.2°C, to 43.4 ± 0.2°C for D131E and 44.0 ± 0.3°C for Q135P.

### 2.2 Arrhythmogenic CaM variants D131E and Q135P show reduced Ca^2+^ binding and altered Ca^2+^/CaM structure and stability

#### 2.2.1 Ca^2+^‐binding affinity

**FIGURE 3:**
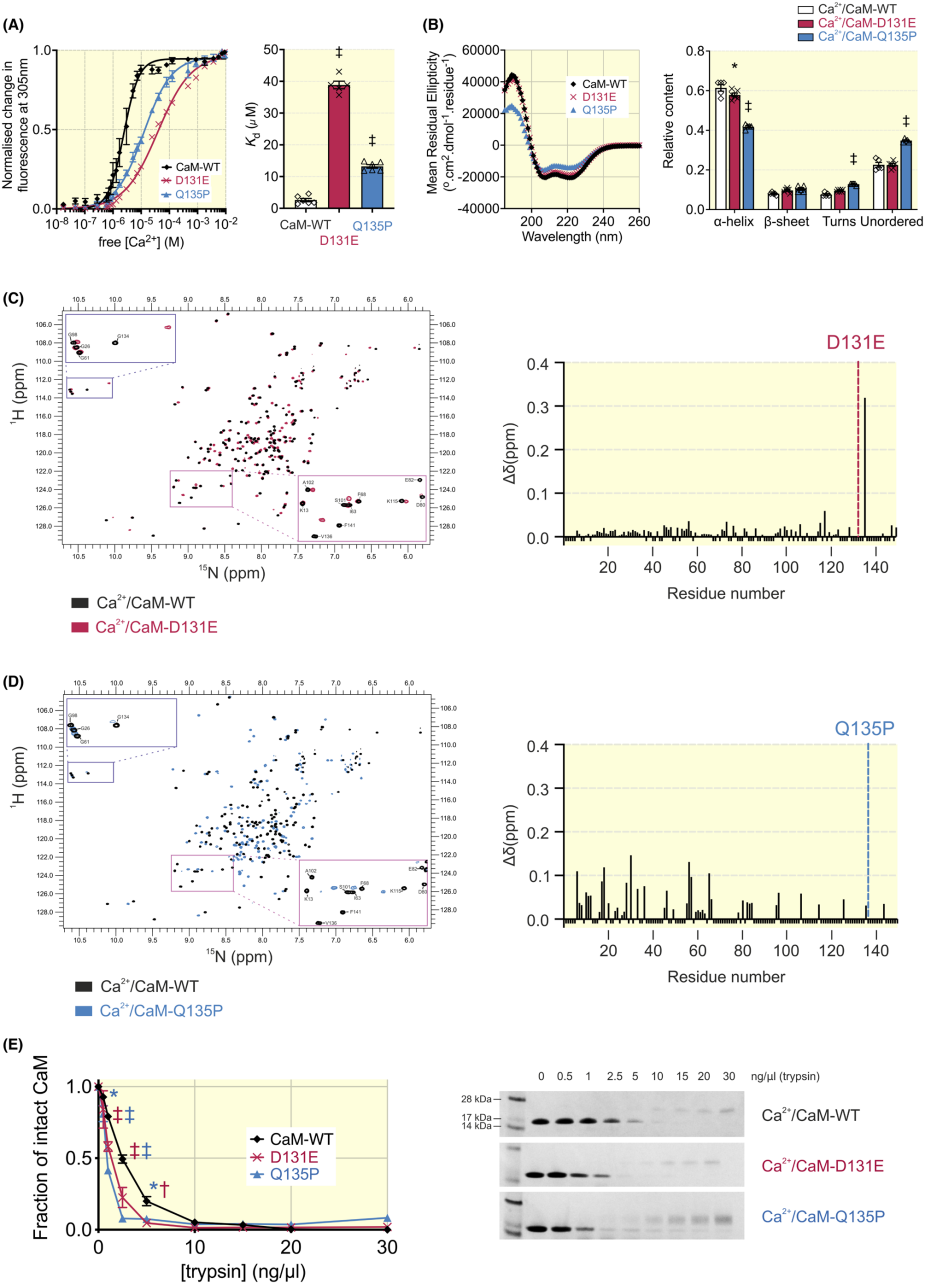
Arrhythmogenic CaM variants D131E and Q135P show decreased binding to Ca^2+^ and altered Ca^2+^‐CaM structure and stability in saturating Ca^2+^ conditions. (A) Measurement of CaM C‐lobe Ca^2+^ affinity by intrinsic tyrosine fluorescence. Data were fitted using the specific binding with Hill Slope function to provide *K*_d_ values and Hill coefficients. Data are presented as mean ± SEM (CaM‐WT, *n* = 6; D131E, *n* = 5; Q135P, *n* = 6) and differences between groups were determined using a one‐way ANOVA with Dunnett's multiple comparisons tests (^‡^ *p* < 0.001). (B) Average far‐UV CD spectra of Ca^2+^‐CaM isoforms and relative secondary structure content estimations based on the CDSSTR prediction algorithm (reference data set 7). Data are presented as mean ± SEM (CaM‐WT, *n* = 5; D131E, *n* = 6; Q135P, *n* = 5) and differences between groups were determined using a two‐way ANOVA with Dunnett's multiple comparisons tests (*p < 0.05, ^‡^ *p* < 0.001). (C,D) Overlays of ^1^H‐^15^N HSQC NMR spectra of Ca^2+^‐CaM‐WT with variants (C) D131E or (D) Q135P and chemical shift analysis. (C,D left panel) Each peak in the spectra represents an amide bond, with its position determined by its chemical shift in the H and N dimensions (x and y axes, respectively). (C,D right panels) Histograms of the chemical shift difference between Ca^2+^‐CaM‐WT and D131E/Q135P variants. For residues that were unable to be assigned, a uniform chemical shift difference value of −0.01 was used. The position of the mutation is illustrated by a colored dotted line. (E) Sensitivity of Ca^2+^‐CaM variants to trypsin degradation using SDS‐PAGE (Coomassie staining). The band at 16.8 kDa representing intact CaM was quantified by densitometry and normalized. Data are presented as mean ± SEM (CaM‐WT, *n* = 4; D131E, *n* = 3; Q135P, *n* = 3) and differences between groups were determined using a one‐way ANOVA with Dunnett's multiple comparisons tests (*p < 0.05, ^†^ *p* < 0.01, ^‡^ *p* < 0.001).

Using tyrosine intrinsic fluorescence spectroscopy (specific for the C‐lobe EF‐hand 3 and 4), we found that both disease‐associated CaM variants have a reduced Ca^2+^‐binding affinity when compared with CaM‐WT (Figure 3A). CaM‐WT had a *K*_d_ for Ca^2+^ of 2.6 ± 0.5 μM, while CaM‐D131E and Q135P showed a 15‐fold decrease (*K*_d_ = 38.9 ± 1.2 μM) and a fivefold decrease (*K*_d_ = 13.2 ± 0.6 μM) in Ca^2+^ affinity, respectively. Additionally, while CaM‐WT demonstrated positive cooperativity of Ca^2+^ binding with a Hill coefficient (*n*) of 2.6 ± 0.6, the disease variants had significantly lower Hill coefficients (*n* = 0.7 ± 0.1 for D131E and *n* = 0.9 ± 0.1 for Q135P).

#### 2.2.2 Structural analysis of Ca^2+^‐bound CaM using CD and NMR

Using CD, we showed that upon Ca^2+^ binding, the relative α‐helical content for CaM‐WT increased from 40 ± 1% to 61 ± 1% (Figure 3B). In Ca^2+^ saturating conditions (1 mM Ca^2+^), CaM‐D131E showed an increase of *α*‐helical content to 58 ± 1%, which is comparable to CaM‐WT. However, for Q135P, the typical increase in α‐helical content associated with the presence of Ca^2+^ was significantly lower (42 ± 1%) with more unordered structures. The reduction in the *α*‐helical content was also observed when using 1 μM Ca^2+^ (Figure S1). To explore the structural implications of mutations in Ca^2+^‐bound CaM further, we used ^1^H‐^15^N HSQC NMR. Chemical shift analysis showed that approximately half of the D131E residues could be assigned, with most of those peaks located in the N‐lobe of the protein (Figure 3C). This indicates that the C‐lobe of CaM D131E is much more severely affected by the mutation than the N‐lobe. However, for Q135P, the fewer residues which could be assigned, showed a greater chemical shift difference when compared with CaM‐WT, suggesting more global perturbation of the structure of CaM. The percentage similarity of ^1^H‐^15^N HSQC NMR spectra was 51.7% for D131E and 12.1% for Q135P, when compared to CaM‐WT. Additionally, there was evidence of heterogeneity in peak intensity for Q135P, indicating reduced protein stability and the presence of multiple conformations.

#### 2.2.3 Trypsin susceptibility of Ca^2+^‐bound CaM variants

To determine the susceptibility to protease digestion of Ca^2+^/CaM, trypsin proteolysis assays were performed in the presence of saturating [Ca^2+^] (Figure 3E). The addition of Ca^2+^ stabilized CaM proteins, as evidenced by the higher concentrations of trypsin required (10 ng/μL trypsin) to completely digest the proteins. We observed that for the disease‐associated CaM variants, the susceptibility to protease digestion was significantly increased, with almost complete degradation at 5 ng/μL trypsin.

### 2.3 CaM D131E shows increased affinity for the IQ domain of Ca_v_1.2

**FIGURE 4:**
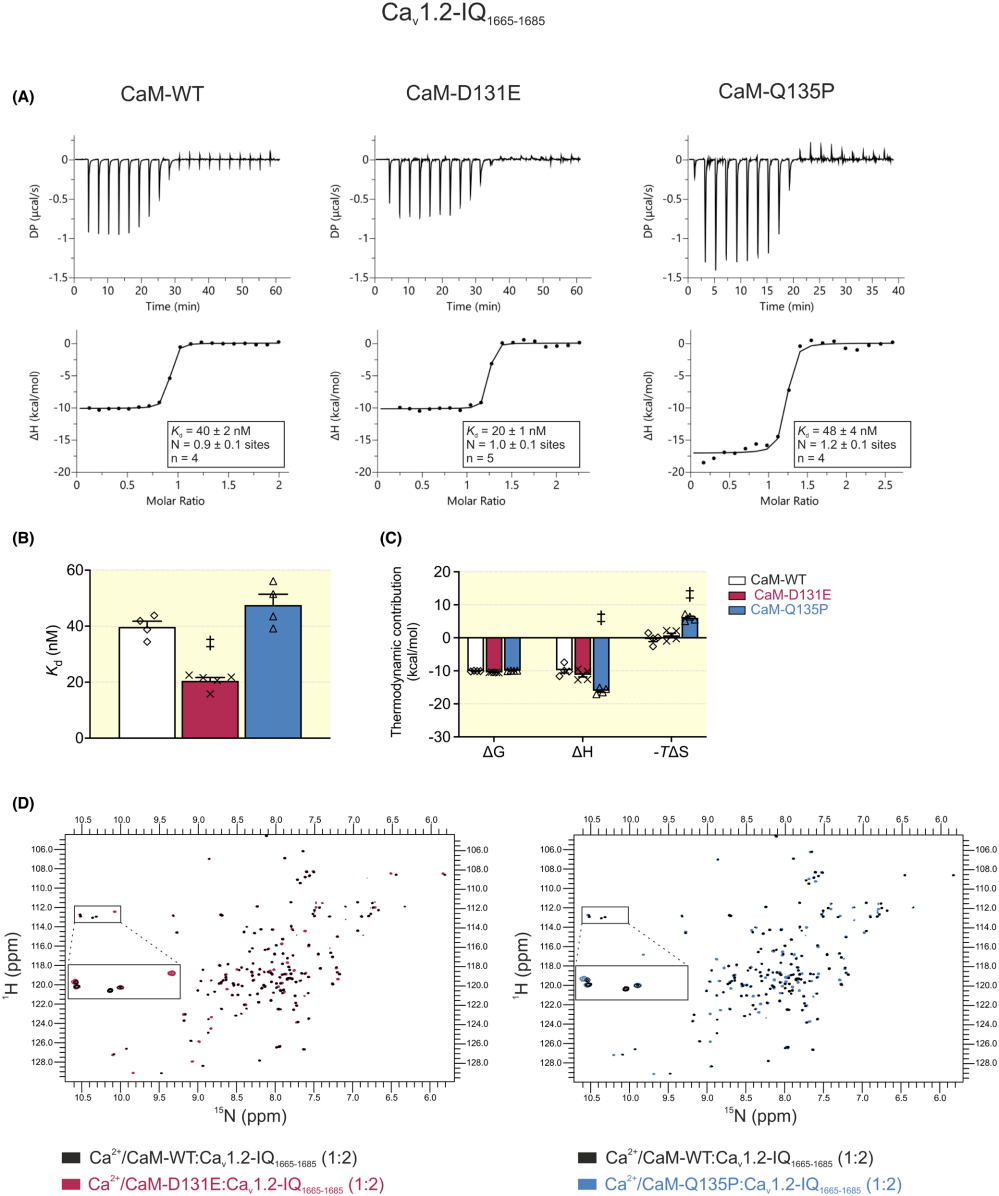
Arrhythmia‐associated CaM variant D131E shows increased binding to Ca_v_1.2‐IQ domain. (A) Representative ITC titration curves, showing the raw heat changes of the interaction (upper panels) and the integration of the isotherm following baseline correction (lower panels). (B) Affinity and (C) thermodynamic profile of the interaction of Ca^2+^/CaM with Ca_v_1.2‐IQ_1665–1685_. Gibbs free energy change (ΔG), enthalpy change (ΔH), and entropy change (‐*T*ΔS). Data are presented as mean ± SEM (CaM‐WT, *n* = 4; D131E, *n* = 5; Q135P, *n* = 4). Experiments were performed at 20°C in Ca^2+^‐saturating conditions (5 mM CaCl_2_). Differences between groups were determined using a one‐way ANOVA (for affinity) and two‐way ANOVA (for thermodynamics) with Dunnett's multiple comparisons tests (^‡^ *p* < 0.001). (D) Overlays of ^1^H‐^15^N HSQC NMR spectra of Ca^2+^‐CaM‐WT:Ca_v_1.2‐IQ_1665–1685_ with variants D131E or Q135P. Each peak in the spectra represents an amide bond, with its position determined by its chemical shift in the H and N dimensions (x and y axes, respectively).

Isothermal titration calorimetry (ITC) was used to measure binding affinity (*K*_d_), stoichiometry (N), and the thermodynamic parameters (enthalpy change, ΔH; change in Gibbs free energy, ΔG; entropy change, ΔS) of Ca^2+^/CaM:Ca_v_1.2‐IQ_1655–1685_ interaction (Figure 4). The stoichiometry of the interaction between Ca^2+^/CaM variants and Ca_v_1.2‐IQ_1655–1685_ was between *N* = 0.9 ± 0.1 and *N* = 1.2 ± 0.1, indicative of a 1:1 binding ratio (Figure 4A). The binding affinity of Ca^2+^/CaM‐Q135P for Ca_v_1.2‐IQ_1655–1685_ was 48 ± 4 nM, which is comparable to CaM‐WT (40 ± 2 nM). However, we observed a significantly lower *K*_d_ for D131E (20 ± 1 nM), indicating a twofold increase in affinity, when compared with CaM‐WT (Figure 4B). All Ca^2+^/CaM:Ca_v_1.2‐IQ_1655–1685_ interactions were energetically favorable and driven by negative enthalpy change (Figure 4C). While the thermodynamic parameters for D131E were comparable to CaM‐WT, we observed a significant change in entropy and enthalpy for the Q135P variant. In the absence of Ca^2+^, the heat changes were too minimal to detect binding between CaM and Ca_v_1.2‐IQ_1655–1685_, indicating that ITC lacked sufficient sensitivity (Figure S2).

In addition, we compared the 3D structure of the Ca^2+^/CaM:Ca_v_1.2‐IQ_1665–1685_ complexes using ^1^H‐^15^N HSQC NMR (Figure 4D). For Ca^2+^/CaM:Ca_v_1.2‐IQ_1655–1685_ complexes, we observed that the NMR fingerprints of the disease‐associated variants D131E and Q135P were more similar to that of CaM‐WT, yet with subtle but notable conformation differences. The percentage similarity of ^1^H‐^15^N HSQC NMR spectra was 85.4% for D131E and 67.1% for Q135P, when compared with CaM‐WT.

### 2.4 CaM Q135P has decreased affinity for the NSCaTE domain of Ca_v_1.2

**FIGURE 5:**
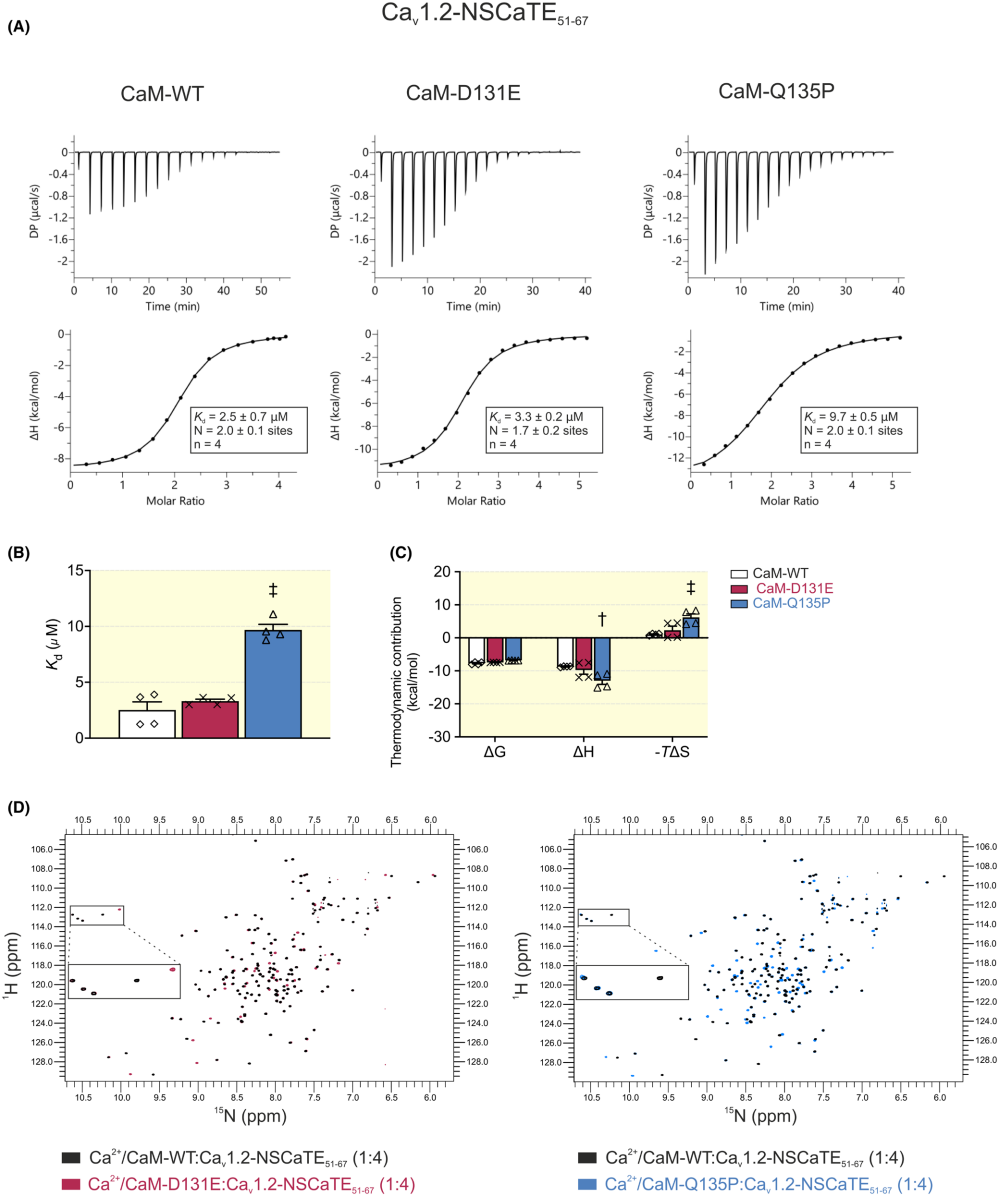
Arrhythmia‐associated CaM variant Q135P shows decreased binding to Ca_v_1.2‐NSCaTE domain. (A) Representative ITC titration curves, showing the raw heat changes of the interaction (upper panels) and the integration of the isotherm following baseline correction (lower panels). (B) Affinity and (C) thermodynamic profile of the interaction of Ca^2+^/CaM with Ca_v_1.2‐NSCaTE_51–67_. Gibbs free energy change (ΔG), enthalpy change (ΔH), and entropy change (‐*T*ΔS). Data are presented as mean ± SEM (CaM‐WT, *n* = 4; D131E, *n* = 4; Q135P, *n* = 4). Experiments were performed at 20°C in Ca^2+^‐saturating conditions (5 mM CaCl_2_). Differences between groups were determined using a one‐way ANOVA (for affinity) and two‐way ANOVA (for thermodynamics) with Dunnett's multiple comparisons tests (^†^ *p* < 0.01, ^‡^ *p* < 0.001). (D) Overlays of ^1^H‐^15^N HSQC NMR spectra of Ca^2+^‐CaM‐WT:Ca_v_1.2‐NSCaTE_51–67_ with variants D131E or Q135P. Each peak in the spectra represents an amide bond, with its position determined by its chemical shift in the H and N dimensions (x and y axes, respectively).

Using ITC, we showed that the Ca^2+^/CaM:Ca_v_1.2‐NSCaTE_51–67_ interaction had a binding stoichiometry between *N* = 1.7 ± 0.2 and *N* = 2.0 ± 0.1, indicating a CaM:NSCaTE_51–67_ binding ratio of 1:2 (Figure 5A). The affinity of CaM D131E for NSCaTE was 3.3 ± 0.2 μM, which is comparable to CaM‐WT (2.5 ± 0.7 μM). However, the *K*_d_ of binding for Q135P was significantly increased to 9.7 ± 0.5 μM (Figure 5B). Interactions for all CaM variants had energetically favorable binding as indicated by negative ΔG and were predominantly enthalpy‐driven (Figure 5C). The variant Q135P showed significant changes in enthalpy and entropy when compared with CaM‐WT. We did not observe any significant binding between CaM and Ca_v_1.2‐NSCaTE_51–67_ in the absence of Ca^2+^ (Figure S3).

In vivo, CaM is pre‐associated with higher affinity binding sites such as the IQ domain in the full Ca_v_1.2 channel complex. Therefore, we examined the interaction between pre‐associated Ca^2+^/CaM:Ca_v_1.2‐IQ_1665–1685_ and Ca_v_1.2‐NSCaTE_51–67_ (Figure S4). Under these conditions, the stoichiometry was determined to be approximately N ~ 1. The affinity was similar for CaM‐WT (2.2 ± 0.2 μM, *n* = 4) and CaM D131E (3.0 ± 0.1 μM, *n* = 4). However, we measured a fivefold increase in *K*_d_ for CaM Q135P (12.3 ± 0.4 μM, *n* = 4), when compared to CaM‐WT. The interaction between Ca^2+^/CaM:Ca_v_1.2‐IQ_1665–1685_ and Ca_v_1.2‐NSCaTE_51–67_ was exothermic, driven by enthalpic contributions, with no significant differences between CaM variants.

Structurally, subtle differences in conformation were observed by ^1^H‐^15^N HSQC NMR of Ca^2+^/CaM‐Ca_v_1.2‐NSCaTE_51–67_ (Figure 5D). The percentage similarity of ^1^H‐^15^N HSQC NMR spectra of Ca^2+^/CaM‐D131E:Ca_v_1.2‐NSCaTE_51–67_ and Ca^2+^/CaM‐Q135P:Ca_v_1.2‐NSCaTE_51–67_ was 86.5% and 72.4%, respectively when compared to Ca^2+^/CaM‐WT:Ca_v_1.2‐NSCaTE_51–67_.

### 2.5 Arrhythmia‐associated mutations impair Ca_v_1.2 Ca^2+^‐dependent inactivation (CDI)

#### 2.5.1 Voltage‐dependence of Ca_v_1.2 activation

**FIGURE 6:**
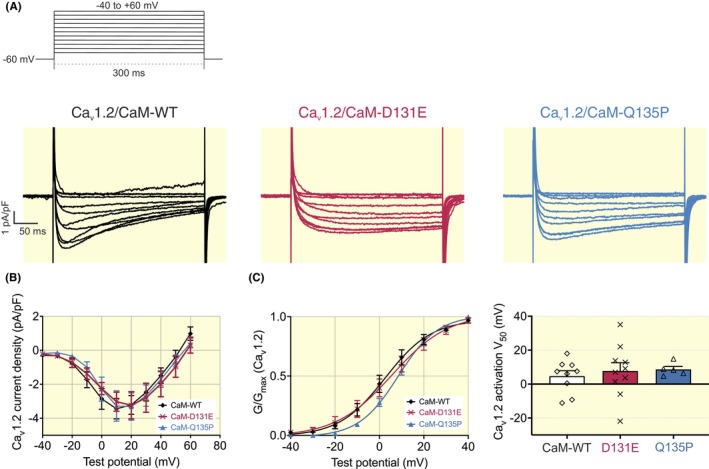
Arrhythmogenic CaM variants do not affect voltage‐dependent activation characteristics of Ca_v_1.2. (A) Voltage step protocol for activation of Ca_v_1.2. Cells were subjected to 300 ms depolarizing steps from −40 to +60 mV in 10 mV increments, from a resting potential of −60 mV. Representative traces from HEK293‐Ca_v_1.2 cells transfected with CaM isoforms, in response to the protocol illustrated in (A). (B) I/V relationships and (C) activation characteristics of Ca_v_1.2 in the presence of CaM variants. Data were normalized to the peak current density for each cell. Channel conductance, G, was normalized to peak conductance, G_max_, to give average activation curves. Half‐maximal activation voltages, V_50_, were calculated from individual curves fitted using the Boltzmann equation. Data are presented as mean ± SEM (CaM‐WT, *n* = 9; D131E, *n* = 10; Q135P, *n* = 5) and differences between groups were determined using a one‐way ANOVA with Dunnett's multiple comparisons tests.

Characteristics of current activation were measured using whole‐cell patch‐clamp electrophysiology in HEK293‐Ca_v_1.2 cells (Figure 6). Representative Ca_v_1.2 current (I_Ca,L_) traces for each CaM variant are displayed in Figure 6A. All CaM variants exhibited a bell‐shaped current–voltage relationship which is characteristic of I_Ca,L_, with depolarizing steps eliciting inward Ca^2+^ currents of increasing amplitude to a peak amplitude at between +10 and +20 mV (Figure 6B). Further depolarization resulted in a decreasing inward current as the test potential approached the current reversal potential. When compared to cells expressing CaM‐WT, no significant difference in maximum peak Ca^2+^ current density was observed in cells expressing the disease‐associated variants D131E and Q135P (−3.6 ± 0.6 pA/pF for CaM‐WT, *n* = 9; −3.5 ± 0.8 pA/pF for CaM‐D131E, *n* = 10; −3.5 ± 0.9 pA/pF for CaM‐Q135P, *n* = 12). Activation curves were fitted to the Boltzmann equation to derive the V_50_ of activation, the voltage at which half‐maximal conductance is reached (Figure 6C). For all CaM variants, activation of Ca_v_1.2 was triggered at ~ −20 mV and reached a maximum conductance at the +40 mV test potential. No significant difference was observed for the V_50_ of activation (4.7 ± 3.2 mV for CaM‐WT; 7.7 ± 4.8 mV for CaM‐D131E; 8.7 ± 1.7 mV for CaM‐Q135P).

#### 2.5.2 Steady‐state inactivation of Ca_v_1.2

To investigate the effect of CaM on the steady‐state inactivation of Ca_v_1.2, the following protocol has been used: from a holding potential of −60 mV, a 1000 ms conditioning pulse ranging from −60 mV to +40 mV was applied to the cell. This was followed by a test pulse to +10 mV, where maximal channel activation occurs. The peak current amplitude at the test potential thus gives an indication of the “available” Ca_v_1.2 whole‐cell current and from this the proportion of Ca_v_1.2 current that has inactivated during the conditioning pre‐pulse can be determined.

**FIGURE 7:**
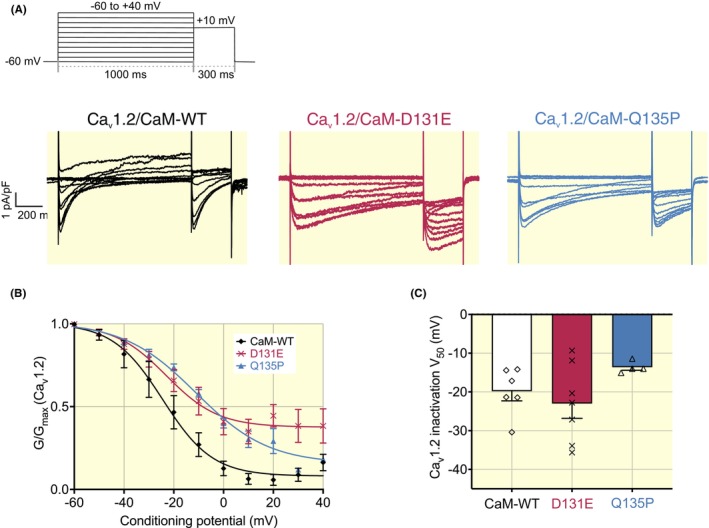
Arrhythmogenic CaM variants do not affect the steady‐state inactivation characteristics of Ca_v_1.2. (A) Voltage step protocol for activation of Ca_v_1.2. Cells were subjected to 1000 ms depolarizing conditioning pre‐pulses from −60 to +40 mV in 10 mV increments, from a resting potential of −60 mV. This was followed by a 300 ms test pulse to +10 mV. Representative traces from HEK293‐Ca_v_1.2 cells transfected with CaM isoforms, in response to the protocol illustrated in (A). (B) I/V relationships and (C) inactivation characteristics of Ca_v_1.2 in the presence of CaM variants. Data were normalized to the peak current density for each cell. Channel conductance, G, was normalized to peak conductance, G_max_, to give average activation curves. Half‐maximal activation voltages, V_50_, were calculated from individual curves fitted using the Boltzmann equation. Data are presented as mean ± SEM (CaM‐WT, *n* = 6; D131E, *n* = 7; Q135P, *n* = 4) and differences between groups were determined using a one‐way ANOVA with Dunnett's multiple comparisons tests.

In cells expressing the CaM variants, we did not observe any significant difference in the steady‐state inactivation of Ca_v_1.2 currents in response to depolarizing pre‐pulses from −60 to +40 mV (V_50_ of inactivation = −19.8 ± 2.5 mV for CaM‐WT; −23.0 ± 3.8 mV for CaM‐D131E; −13.6 ± 0.8 mV for CaM‐Q135P) (Figure 7). Even with the prolonged depolarizing step utilized in the inactivation protocol, Ca^2+^ currents for the disease‐associated CaM variants failed to fully inactivate.

#### 2.5.3 Ca^2+^‐dependent inactivation (CDI) of Ca_v_1.2

To assess the effects of the CaM variants on CDI we used the following protocol: from a holding potential of −60 mV, a depolarizing pulse to +10 mV was applied to the cell to induce a maximal Ca_v_1.2 current, which subsequently inactivated during the 300 ms duration of the pulse. The extracellular bath solution was then exchanged from one containing 2 mM CaCl_2_ to one containing 2 mM BaCl_2_ and the protocol repeated. Ba^2+^ is able to influx through the Ca_v_1.2 pore but cannot bind to CaM to induce channel inactivation. This protocol was also repeated for test potentials ranging from −40 mV to +20 mV.

**FIGURE 8:**
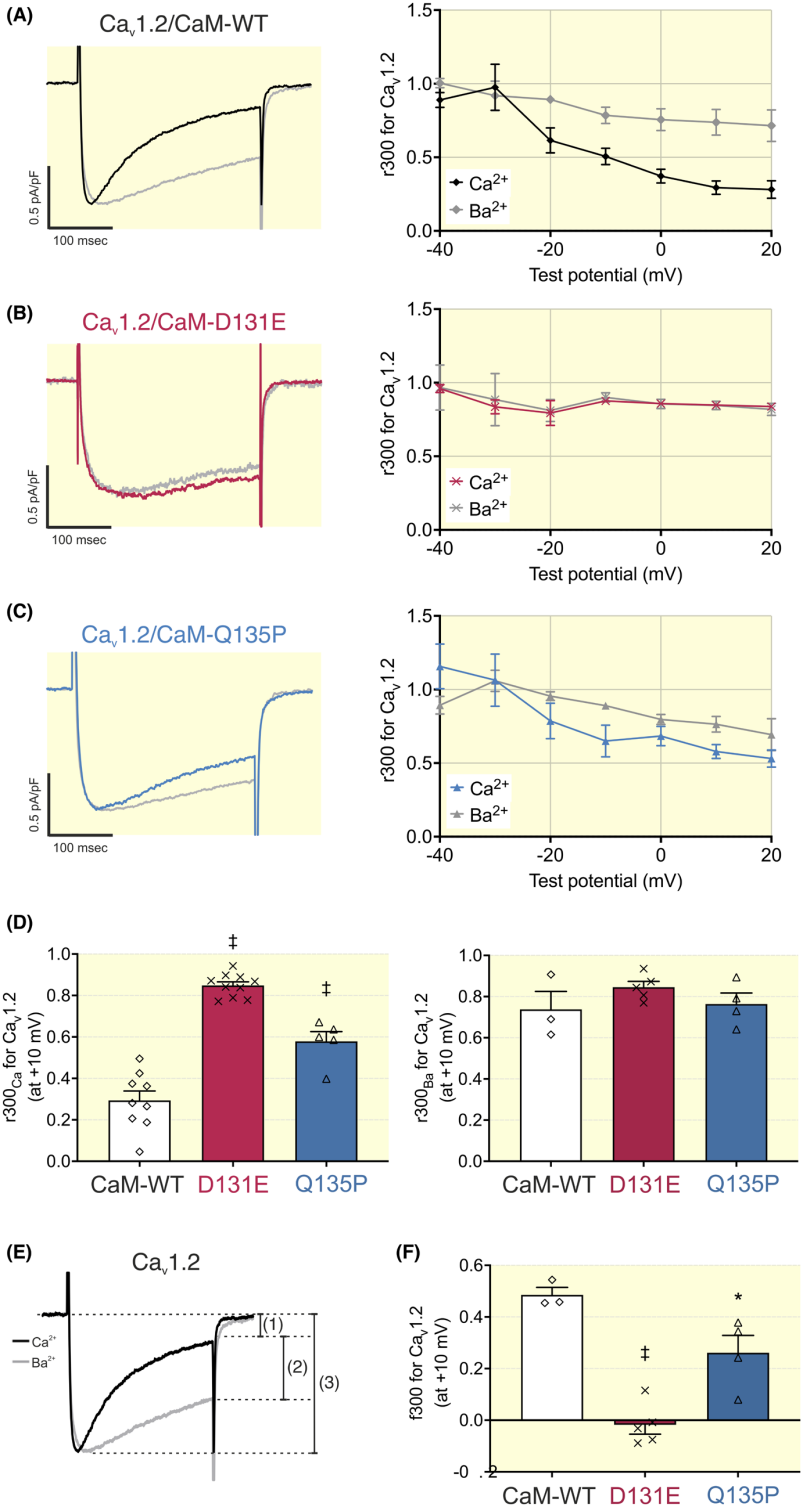
Arrhythmogenic CaM variants severely disrupt Ca^2+^‐dependent inactivation (CDI) of Ca_v_1.2. (Left panels) Representative Ca^2+^ and Ba^2+^ current traces from HEK293‐Ca_v_1.2 cells transfected with (A) CaM‐WT, (B) D131E or (C) Q135P, in response to a 300 ms pulse to +10 mV, normalized to their respective peak current. (Right panels) Fractional residual Ca^2+^ and Ba^2+^ current at the end of the 300 ms pulse (r300), at test potentials ranging from −40 to +20 mV. Data are presented as mean ± SEM (CaM‐WT Ca^2+^ *n* = 9, Ba^2+^ *n* = 3; D131E Ca^2+^ *n* = 10, Ba^2+^ *n* = 5; Q135P Ca^2+^ *n* = 5, Ba^2+^ *n* = 4). (D) Residual Ca^2+^ and Ba^2+^ currents at the end of a 300 ms pulse (r300_Ca_ and r300_Ba_, respectively), at +10 mV. Data are presented as mean ± SEM (CaM‐WT Ca^2+^ *n* = 9, Ba^2+^ *n* = 3; D131E Ca^2+^ *n =* 10, Ba^2+^ *n* = 5; Q135P Ca^2+^ *n* = 5, Ba^2+^ *n* = 4) and differences between groups were determined using a one‐way ANOVA with Dunnett's multiple comparisons tests (^‡^ *p* < 0.001). (E) Representative normalized Ca^2+^ (black) and Ba^2+^ (gray) currents from HEK923‐Ca_v_1.2 cells in response to a 300 ms depolarizing pulse to +10 mV: R300_Ca_ are calculated as the residual Ca^2+^ current at the end of the pulse (1) normalized to the corresponding peak current (3). r300_Ba_ is calculated as the residual Ba^2+^ current at the end of the pulse (2) normalized to the corresponding peak current (3). f300 is the difference between r300_Ba_ and r300_Ca_, normalized to r300_Ba_, and represents the proportion of inactivation due to the presence of Ca^2+^ (CDI). (F) Proportion of inactivation due to CDI (f300), at +10 mV. Data are presented as mean ± SEM (CaM‐WT *n* = 3; D131E *n* = 5; Q135P *n* = 4) and differences between groups were determined using a one‐way ANOVA with Dunnett's multiple comparisons tests (*p < 0.05, ^‡^ *p* < 0.001).

The residual current at the end of the test pulse (r300_Ca_) was compared with when Ba^2+^ was used as the charge carrier (r300_Ba_). This allows us to separate and discriminate Ca^2+^‐dependent from Ca^2+^‐independent inactivation. For cells expressing both disease‐associated variants, CDI of Ca_v_1.2 was significantly impaired when compared with cells expressing CaM‐WT and we observed a gradual decrease in r300 upon increasing depolarization for all CaM variants (Figure 8). CaM‐WT demonstrated the greatest decrease in r300_Ca_ with depolarization, with large differences between r300_Ca_ and r300_Ba_ evident at voltages above 0 mV. Conversely, CaM variant overexpression resulted in larger r300_Ca_ values much closer to their equivalent r300_Ba_ values, particularly for CaM‐D131E. At +10 mV, the residual Ba^2+^ current at the end of the test pulse (r300_Ba_) was comparable for all CaM variants (0.74 ± 0.09 for CaM‐WT; 0.85 ± 0.03 for CaM‐D131E; 0.76 ± 0.05 for CaM‐Q135P), indicating no difference in Ca^2+^‐independent inactivation (Figure 8D). In the presence of Ca^2+^, r300_Ca_ values were significantly increased from 0.29 ± 0.05 (CaM‐WT) to 0.85 ± 0.02 (CaM‐D131E) and 0.56 ± 0.05 (CaM‐Q135P). The difference between r300_Ca_ and r300_Ba_, normalized to r300_Ba_, indicates the proportion of total inactivation due to CDI, f300 (Figure 8E). CDI was decreased for cells expressing CaM‐Q135P (f300 = 0.26 ± 0.07) and completely abolished for CaM‐D131E (f300 = −0.02 ± 0.04) when compared to CaM‐WT (f300 = 0.49 ± 0.03) (Figure 8F).

## 3 DISCUSSION

LQTS and CPVT are inherited cardiac disorders which have been associated with CaM dysfunction (see reviews^9^, ^10^, ^11^). CaM is a Ca^2+^‐binding protein which plays a crucial role in modulating ion channels that regulate the cardiac action potential. Mutations in CaM, particularly in EF‐hands, have the potential to affect not only the capacity for CaM to bind Ca^2+^, but also how CaM interacts with and regulates key ion channels involved in the ventricular action potential. This would create an environment conducive to potentially life‐threatening arrhythmias. In this multidisciplinary study, we showed that the arrhythmia‐associated EF‐hand 4 mutations (D131E and Q135P) affect the structure–function relationship of CaM and the subsequent regulation of the Ca_v_1.2 channel. Table 1 and Table 2 summarize the major biophysical and functional properties of arrhythmogenic CaM variants D131E and Q135P determined in this study, respectively.

**TABLE 1:** Summary of binding affinities of LQTS‐associated CaM variants for Ca^2+^ and Ca_v_1.2 CaMBDs.

	Ca^2+^	Ca_v_1.2‐IQ	Ca_v_1.2‐NSCaTE	Ca_v_1.2‐NSCaTE (pre‐bound Ca_v_1.2‐IQ)
CaM‐WT	2.6 ± 0.5	0.040 ± 0.002	2.5 ± 0.7	2.2 ± 0.2
D131E	38.9 ± 1.2^‡^	0.020 ± 0.001^‡^	3.3 ± 0.2	3.0 ± 0.1
Q135P	13.2 ± 0.6^‡^	0.048 ± 0.004	9.7 ± 0.5^‡^	12.3 ± 0.4^‡^

*Note*: *K*_d_ values (μM), mean ± SEM.

^‡^ *p* < 0.001, vs. CaM‐WT.

**TABLE 2:** Summary of functional effects of LQTS‐associated CaM variants on Ca_v_1.2 activity.

	Ca_v_1.2 (patch‐clamp electrophysiology)				
	Peak current density (pA/PF)	Reversal potential (mV)	V_50_ activation (mV)	V_50_ inactivation (mV)	CDI (f300)
CaM‐WT	−3.6 ± 0.6	52.33 ± 2.8	4.7 ± 3.2	−19.8 ± 2.5	0.49 ± 0.03
D131E	−3.5 ± 0.8	53.8 ± 2.6	7.7 ± 4.8	−23.0 ± 3.8	−0.02 ± 0.04^‡^
Q135P	−3.5 ± 0.9	54.0 ± 3.6	8.7 ± 1.7	−13.6 ± 0.8	0.26 ± 0.07*

*Note*: Values are mean ± SEM.

*p < 0.05, ^‡^ *p* < 0.001, vs. CaM‐WT.

In the apo state, CaM adopts a compact and yet highly flexible conformation.^28^, ^45^ For the disease‐associated CaM variants (D131E and Q135P), we did not see major secondary and tertiary structural changes when compared with apo/CaM‐WT, as observed for other variants.^46^, ^47^, ^48^, ^49^, ^50^, ^51^ However, we found subtle but significant differences in temperature sensitivity, indicative of an alteration in the strength of intramolecular interactions within CaM.

It has been shown that binding to Ca^2+^ to both C‐ and N‐lobes increases the stability of CaM and causes dramatic changes in its structure, exposing hydrophobic pockets to allow interactions with CaMBDs on target proteins.^25^, ^52^, ^53^, ^54^ We found a significantly reduced C‐lobe Ca^2+^ binding affinity for CaM D131E and Q135P, as previously observed.^14^ Affinity for Ca^2+^ was reduced 15‐fold and 5‐fold for CaM D131E and Q135P, respectively. In addition, we showed a loss of Ca^2+^ binding cooperativity in the disease‐associated CaM variants, indicating that mutations in EF‐hand 4 can affect EF‐hand 3 Ca^2+^ interactions.

In terms of structure, we demonstrated a reduced α‐helical content for Ca^2+^/CaM D131E and even more so for Q135P. This is in agreement with data for other variants.^44^, ^50^, ^51^ While other arrhythmia‐associated variants show conformational changes localized to the point of mutation,^46^, ^49^, ^51^, ^55^ our data from ^1^H‐^15^N HSQC NMR and trypsin digestion we found global changes in the tertiary structure and stability. This indicates global alterations to conformation and folding, with decoupling of Ca^2+^‐induced conformational change, and highlights the crucial role of EF‐hand 4 Ca^2+^‐coordinating residues as modulators of overall CaM structure and function. This is in accordance with proteolysis and dynamic light scattering data obtained for D95H/V, D129G and D131E/H, and NMR data for D131H.^22^, ^55^, ^56^ Our results therefore demonstrate that while mutations have subtle effects on apo/CaM, they severely alter Ca^2+^‐bound CaM stability, structure, and Ca^2+^ binding at the C‐lobe. This would have consequences on the ability of CaM to sense [Ca^2+^] changes, leading to dysfunctional cardiac ion channel regulation.

Ca_v_1.2 plays a pivotal role in cardiac contraction. Opening of Ca_v_1.2 channels and the subsequent increase in intracellular Ca^2+^ concentration (via CICR) are fundamental events in excitation–contraction coupling. This process allows the connection of the electrical activity of the heart to the mechanical contraction of the cardiac muscle. It has been shown that CaM interacts with the cytoplasmic C‐terminal tail of Ca_v_1.2, influencing its function and regulating Ca^2+^ influx into cells upon membrane depolarization.^34^, ^35^, ^37^, ^39^, ^40^, ^41^, ^42^, ^57^, ^58^, ^59^, ^60^, ^61^, ^62^, ^63^, ^64^, ^65^, ^66^ The main CaMBD on the C‐terminal of the channel is the IQ domain, which has been shown to interact with CaM at various Ca^2+^ concentrations.^39^, ^40^, ^41^, ^42^, ^50^, ^55^, ^67^ However, ITC was not sensitive enough to detect CaM binding to Ca_v_1.2‐IQ at low Ca^2+^ concentrations. Here we showed that Ca^2+^/CaM binds to the Ca_v_1.2‐IQ domain with a *K*_d_ of 40 ± 2 nM with a 1:1 stoichiometry, similar to previously reported values.^35^, ^36^, ^41^, ^42^, ^43^, ^50^, ^55^, ^61^, ^62^ This interaction is crucial for priming the channel for rapid responses to Ca^2+^ influx when the channel is activated. While Q135P did not show any difference in *K*_d_, we found a significant twofold increase in affinity for Ca^2+^/CaM D131E for Ca_v_1.2‐IQ. This is a unique feature of D131E (and E140G^50^) as arrhythmia‐associated CaM mutations generally have reduced binding to Ca_v_1.2‐IQ in the presence of Ca^2+^.^44^, ^46^, ^55^, ^68^, ^69^ As the heterozygous variants D131E and Q135P only affect 1/6 of the CaM alleles,^14^ disease‐affected patients will present a mixture of CaM‐WT and the arrhythmogenic variant. The higher affinity interaction between CaM‐D131E and Ca_v_1.2‐IQ could result in the blockade of CaM‐WT binding at this site.

Ca^2+^/CaM also binds to the Ca_v_1.2‐NSCaTE region on the N‐terminal of Ca_v_1.2 with an affinity in the high nM to low μM range.^36^, ^37^, ^38^, ^43^, ^50^ It has been demonstrated that binding to both Ca_v_1.2‐IQ and Ca_v_1.2‐NSCaTE domains is crucial to the mechanism of CaM‐mediated CDI in Ca_v_1.2. In our study, we show that the binding of CaM‐WT to the Ca_v_1.2‐NSCaTE domain was weaker than to Ca_v_1.2‐IQ (*K*_d_ of 2.5 ± 0.7 μM compared with 40 ± 2 nM). This is in agreement with previous studies that calculate *K*_d_ for Ca^2+^/CaM‐WT:NSCaTE in the range of 0.6–2.9 μM.^36^, ^38^, ^43^, ^50^ We observed a binding ratio of 1:2 for Ca^2+^/CaM:Ca_v_1.2‐NSCaTE, as observed by other groups.^38^, ^43^, ^50^ However, this observation is unlikely to be physiologically relevant as CaM would be pre‐associated to Ca_v_1.2‐IQ and therefore would present a stoichiometry of binding with Ca_v_1.2‐NSCaTE of only N ~ 1. Using ITC, we showed that pre‐associated CaM:Ca_v_1.2‐IQ interacted with Ca_v_1.2‐NSCaTE with a molar ratio of 1:1, as previously shown.^50^ We demonstrated that the affinity for Ca_v_1.2‐NSCaTE is still significantly decreased for the arrhythmia‐associated variant Q135P, from 2.2 ± 0.2 μM (CaM‐WT) to 12.3 ± 0.4 μM (CaM‐Q135P). The significant reduction in binding to Ca_v_1.2‐NSCaTE was also observed for other variants, such as the LQTS‐associated variant CaM E140G.^50^

For the first time, we reveal the effect of the D131E and Q135P mutations on the interaction with the NSCaTE domain of Ca_v_1.2. While D131E did not show any difference in *K*_d_, we found a significant, ~ 5‐fold decrease in affinity for Ca^2+^/CaM Q135P for Ca_v_1.2‐NSCaTE; reduced interaction with this site has also been observed for E140G.^50^, ^70^ As NSCaTE is an effector site for CaM, it is theorized to play a vital role in CDI.^37^, ^38^ This loss of interaction for Q135P could therefore lead to a reduction in CDI, increasing the risk of arrhythmogenic Ca^2+^ influx.

**FIGURE 9:**
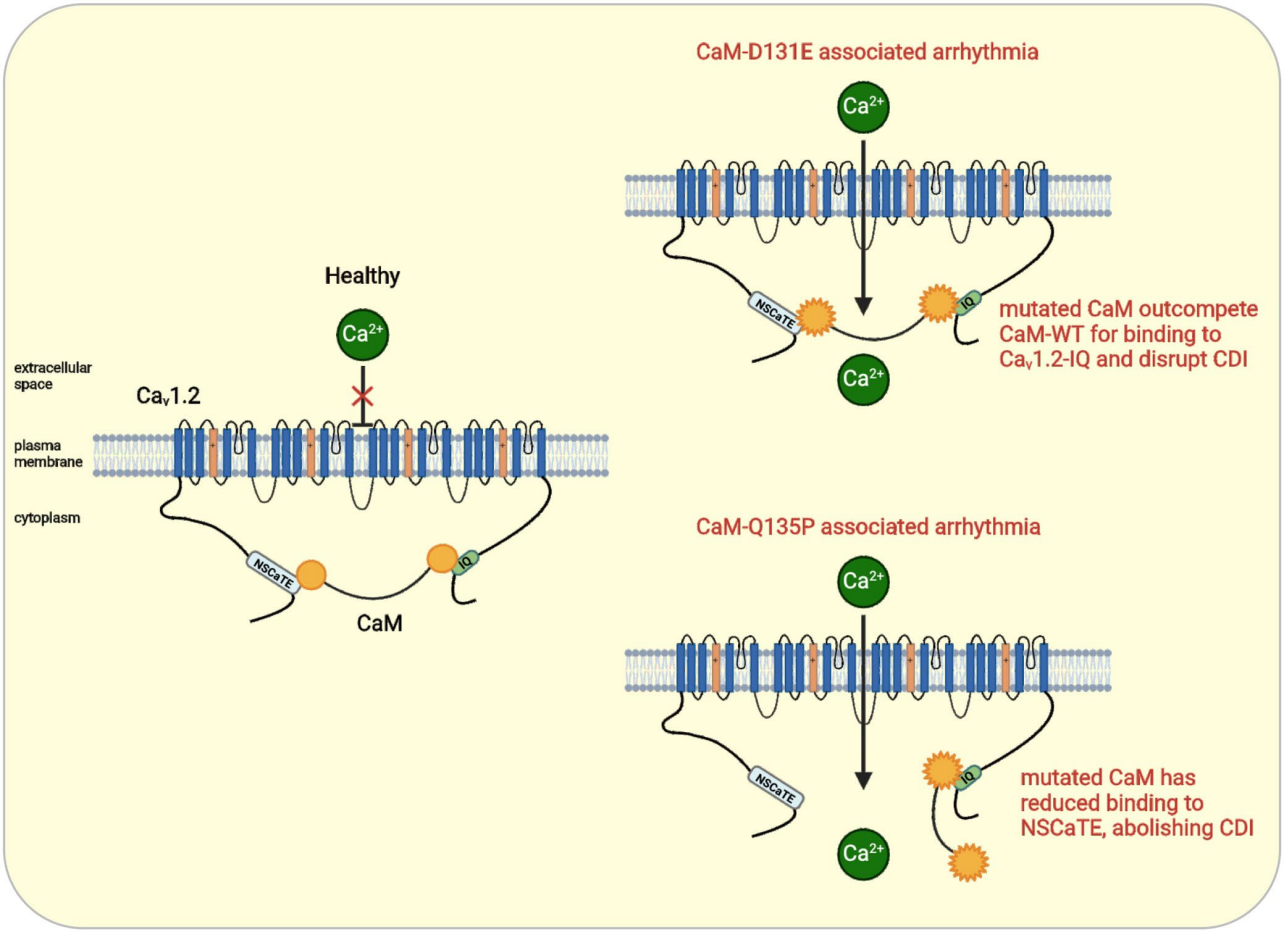
Regulation mechanism of Ca_v_1.2 by arrhythmia‐associated CaM variants D131E and Q135P. In normal conditions, Ca_v_1.2 opening elicited by an action potential triggers Ca^2+^ entry into the cardiomyocyte. CaM rapidly senses Ca^2+^ influx and when intracellular [Ca^2+^] increases, CaM facilitates Ca^2+^‐dependent inactivation (CDI) by bridging the C‐terminal and N‐terminal domains of the channel. This prevents further Ca^2+^ influx and protect the cell from Ca^2+^ overload. In arrhythmia, CaM affinity for Ca^2+^ is reduced, therefore intracellular [Ca^2+^] changes are not sensed appropriately and Ca_v_1.2 remains open longer. Additionally, CaM has a higher affinity for Ca_v_1.2‐IQ, suggesting it may outcompete CaM‐WT on the C‐terminal domain of the channel, and a reduced binding affinity for the NSCaTE domain, which will disrupt CaM‐dependent CDI. Altogether, this would promote excessive Ca^2+^ entry into the cell, and disrupted Ca^2+^ handling would contribute to the arrhythmia phenotype. Created in BioRender. Helassa, N. (2024) BioRender.com/m99v569.

All CaM isoforms demonstrated energetically favorable binding to Ca_v_1.2 CaMBDs as indicated by negative ΔG and were predominantly enthalpy‐driven. This suggests that binding mainly consisted of hydrogen bond formation, although Q135P displayed altered changes in entropy, suggesting a loss of hydrophobic interaction. In addition to the effects on binding thermodynamics and affinity, both disease‐associated CaM variants showed altered conformation when bound to Ca_v_1.2 CaMBDs. This may affect the transduction of Ca^2+^ signals by CaM to the channel, even when the binding to the CaMBD on the channel is not affected. As the IQ domain is theorized to have a greater contribution to Ca_v_1.2 CDI than the NSCaTE region, these differences in biophysical and binding properties could correlate with the time of onset of the disease and the severity of the phenotype.^14^, ^37^ Here, we present a basis for Ca_v_1.2 dysregulation in arrhythmia, where D131E can outcompete CaM‐WT for binding to the IQ domain of Ca_v_1.2. This thereby prevents CaM‐WT from binding and contributes to a defective Ca_v_1.2 inactivation in response to elevated Ca^2+^ levels. Persistent opening of Ca_v_1.2 would disrupt the finely tuned ionic balance required for cardiac cell repolarization and increase the risk of arrhythmias. On the other hand, the weaker interaction of Q135P with the Ca_v_1.2‐NSCaTE region may also contribute to the functional effects of the mutation. As NSCaTE is believed to interact with only the N‐lobe of CaM in vivo, while the C‐lobe remains tethered to the C terminus of the channel,^37^ it is surprising that the Q135P mutation (located in the C‐lobe of CaM) disrupts the interaction with NSCaTE. However, our NMR data demonstrate that global structural rearrangements occur for Q135P, which could alter CaM binding with interacting partners at for both N‐ and C‐lobe. Weaker binding to NSCaTE would disrupt Ca_v_1.2 inactivation, leading to an increase in intracellular Ca^2+^ concentration and promoting arrhythmogenic activity. The proposed mechanism underscores the importance of a balanced CaM‐Ca_v_1.2 interaction for proper electrophysiological function and highlights how specific mutations can tip this balance toward pathological outcomes (Figure 9).

It has been shown that disruption of Ca_v_1.2 activity has profound effects on the rhythmic contraction of the heart^71^, ^72^ and a number of missense mutations in Ca_v_1.2 have been associated with cardiac arrhythmia.^73^, ^74^, ^75^, ^76^ Ca_v_1.2 inactivation is under the tight control of two separate processes: voltage‐dependent inactivation^77^, ^78^ and Ca^2+^‐dependent inactivation mediated by CaM.^31^ CaM interaction with the IQ and NSCaTE domains of Ca_v_1.2 plays an important role in CDI.^37^, ^59^, ^79^ CaM facilitates a negative feedback mechanism by sensing [Ca^2+^]_cyt_ increase, leading to a conformational change allowing bridging the N‐ and C‐termini of Ca_v_1.2, resulting in channel inactivation.^37^ Therefore, mutations that disturb interaction between CaM and CaMBDs of Ca_v_1.2 are likely to detrimentally affect CDI, leading to prolonged Ca^2+^ influx. For arrhythmogenic CaM variants D131E and Q135P, we did not observe significant differences in Ca_v_1.2 current densities, or voltage‐dependence of channel activation/inactivation. This is in accordance with previous studies on other CaM variants.^17^, ^19^, ^21^, ^22^, ^50^, ^80^, ^81^, ^82^ However, we revealed a clear Ca^2+^‐dependent effect of CaM mutations on the inactivation of Ca_v_1.2 channels with a dramatic impairment in CDI. D131E induced the most severe effects on inactivation with CDI essentially abolished. The difference in the severity of CDI between the variants could be explained by the stronger disruption of Ca^2+^ binding in combination with the altered interaction with the IQ domain for D131E. Conversely, Q135P showed a more moderate reduction in Ca^2+^ affinity and reduced interaction with the NSCaTE region of the channel, which is known to contribute less to CDI than the IQ domain. While the severity of CDI impairment does to some extent correlate with the age of clinical presentation of disease, with D131E causing neonatal LQTS versus Q135P only eliciting evidence of cardiac arrhythmia at the age of 8 years old,^14^ this may not be an accurate indicator of disease severity. Our data points toward a potential common pathophysiological mechanism involving disruption of Ca_v_1.2 inactivation, where ventricular action potential duration in cardiomyocytes is increased, affecting the myocyte action potential waveform and leading to an increased risk of early after‐depolarizations (Figure 9).

While we demonstrate the detrimental effects of arrhythmia‐associated CaM mutations on Ca_v_1.2 function, the extensive number of CaM targets in cardiac ventricular myocytes suggests that other CaM‐regulated proteins could be affected. Altered interactions with other targets could ameliorate or aggravate the proarrhythmic effects, contributing to differences in clinical severity. D131E and Q135P have been associated with a mixed LQTS/CPVT phenotype.^14^ CPVT is most commonly attributed to RyR2 dysfunction. It has already been demonstrated that these mutations negatively impact CaM binding to RyR2^83^ and for many CaM variants including D131E and Q135P, RyR2 activation thresholds were altered.^14^, ^19^, ^84^, ^85^ This could lead to compromised RyR2 and Ca_v_1.2 functions. It is also possible that other CaM‐regulated components of cardiac excitability such as Na_v_1.5 and K_v_7.1 are affected. For example, there is evidence that CaM‐N137K enhanced K_v_7.1 activity could counter increased Ca_v_1.2 activity.^48^ This would cause a reduction of the overall impact on the ventricular action potential duration and result in a milder phenotype.^48^ On the other hand, the reduced binding to Na_v_1.5 exhibited by CaM‐G113R/W may lead to an increased persistent Na^+^ current, which in combination with the mild disruption of Ca_v_1.2 and RyR2 may explain the severe phenotype seen in patients with these mutations.^69^, ^86^ Ergo, interaction of these CaM variants with other cardiac targets will be necessary to better understand the impact of the mutations at the myocyte and organism level.

## 4 CONCLUSION

In this study, we demonstrated that for arrhythmia‐associated CaM variants D131E and Q135P, reduced Ca^2+^ binding affinity, global alterations of Ca^2+^/CaM conformation, and changes to the interaction with the Ca_v_1.2 channel are all significant factors which differentially contribute to the severe loss of CDI. This would contribute to the prolongation of the action potential duration in cardiomyocytes and increase the proarrhythmic risks of Ca^2+^ overload and early‐ after‐depolarizations (EAD), characteristic of the arrhythmia phenotype observed in patients with these mutations. We provide a vital contribution to the understanding of calmodulinopathies, with insights into the molecular basis of inherited cardiac arrhythmia syndromes associated with CaM mutations.

## 5 MATERIALS AND METHODS

### 5.1 Molecular Biology

For recombinant CaM protein expression, site‐directed mutagenesis (SDM) was performed on pE‐SUMOPro‐CaM^49^ to generate the equivalent LQTS‐associated variants D131E and Q135P constructs, following the QuikChange® protocol (Agilent Technologies).

For mammalian expression of CaM in electrophysiological studies, CaM variants were subcloned from the pE‐SUMOPro vector into pHIV‐IRES‐EGFP vector using NEBuilder HiFi DNA Assembly. The pHIV‐IRES‐EGFP plasmid construct was a gift from Bryan Welm and Zena Werb (Addgene plasmid # 21373).^87^ These pHIV‐CaM‐IRES‐EGFP constructs allowed for the co‐expression of CaM and the fluorescent marker EGFP as two distinct proteins, under the control of the same promoter. Primers used are listed in Table 3 and all constructs generated were verified by Sanger sequencing (MRC PPU, University of Dundee, UK).

**TABLE 3:** Primer sequences used in this study.

	Primer	Sequence (5′‐3′)
pE‐SUMOPro‐CaM SDM	D131E fwd	AAATGATCAGGGAAGCAGATATTGAGGGTGATGGTCAAG
	D131E rev	CTTGACCATCACCCTCAATATCTGCTTCCCTGATCATTT
	Q135P fwd	GCAGATATTGATGGTGATGGTCCAGTAAACTATGAAGAGTTTGTA
	Q135P rev	TACAAACTCTTCATAGTTTACTGGACCATCACCATCAATATCTGC
pHIV‐CaM‐IRES‐EGFP	pHIV fwd	AGGATCCGCCCCTCTCCC
	pHIV rev	CGCTCACGACACCTGAAATG
	CaM fwd	CATTTCAGGTGTCGTGAGCGATGGCTGACCAACTGACTG
	CaM rev	GAGGGAGAGGGGCGGATCCTTCACTTTGCTGTCATCATTTG

### 5.2 Recombinant CaM protein expression and purification

Recombinant CaM protein variants were expressed and purified as described previously.^49^, ^50^, ^51^ Briefly, pE‐SUMOPro‐CaM expression vectors were transformed into *Escherichia coli* BL21(DE3) STAR‐competent cells. Cells were cultured in 2xYT media supplemented with 100 μg/mL kanamycin, and incubated at 37°C, 200 rpm. Recombinant CaM overexpression was induced by the addition of 0.5 mM μM Isopropyl *β*‐D‐1‐thiogalactopyranoside once the optical density of the bacterial culture at 600 nm reached between 0.5 and 1. The culture was then allowed to grow for 18 h at 18°C, 180 rpm. Cells were pelleted by centrifugation and resuspended in 50 mM HEPES, 200 mM NaCl, pH 7.5, supplemented with Proteoloc Protease Inhibitor Cocktail (Abcam). Cells were lysed by 30 min incubation with lysozyme (1 mg/mL) followed by sonication, on ice. Lysates were clarified by the addition of 2 μL BaseMuncher endonuclease (Abcam) and ultracentrifugation at 100000 *g*, 4°C, for 1 h. Clarified lysates were loaded onto a HisTrap HP 5 mL column (GE Healthcare) equilibrated with 50 mM HEPES, 200 mM NaCl, pH 7.5. CaM protein was eluted from the column with a 10–500 mM imidazole linear gradient. Eluted fractions were dialyzed overnight at 4°C, using SnakeSkin™ Dialysis Tubing membrane (Thermo Scientific) with a 3.5 kDa molecular weight cut‐off. The SUMO tag was removed by incubation with SUMO protease for 1 h at 30°C (1:2000 protease:CaM molar ratio). Samples were then further purified using HisTrap to remove the cleaved tag and size exclusion chromatography (HiLoad 16/600 Superdex 75 pg., GE Healthcare) on an ÄKTA Pure system at 4°C, equilibrated with 20 mM HEPES, 50 mM NaCl, pH 7.5. Purified CaM protein was concentrated and aliquoted, flash frozen in liquid nitrogen, and stored at −80°C.

For NMR experiments,^15^ N labeled CaM protein was generated by culturing transformed BL21(DE3) STAR cells in minimal medium containing 88 mM Na_2_HPO_4_, 55 mM KH_2_PO_4_, 30 μM thiamine‐HCl, 136 μM CaCl_2_·2H_2_O, 1 mM MgSO_4_·7H_2_O, 19 mM^15^ NH_4_Cl (Cambridge Isotope Laboratories) and 22 mM glucose. Induction, cell lysis, and protein purification were performed as described above.

### 5.3 Peptides

Peptide sequences corresponding to CaMBDs on the Ca_v_1.2 *α*_1C_ subunit were synthesized and HPLC purified (>95%) by GenicBio:

- NSCaTE (Ca_v_1.2‐NSCaTE_51‐67_, SWQAAIDAARQAKLMGS)
- IQ (Ca_v_1.2‐IQ_1665‐1685_, KFYATFLIQEYFRKFKKRKEQ).

### 5.4 Quantification of protein and peptide concentrations

CaM protein, NSCaTE_51–67_ and IQ_1665–1685_ peptide concentrations were calculated based on absorbance at 280 nm, using a DeNovix DS‐11+ Spectrophotometer. Extinction coefficients (ɛ) for the CaMBD peptides and CaM protein were calculated using the ExPASy ProtParam tool^88^: ε (CaM) = 2980 M^−1^ cm^−1^, ε (His‐SUMO‐CaM) = 4470 M^−1^ cm^−1^, ε (Ca_v_1‐2‐NSCaTE_51–67_) = 5500 M^−1^ cm^−1^, ε (Ca_v_1.2‐IQ_1665–1685_) = 2980 M^−1^ cm^−1^.

### 5.5 Fluorescence spectroscopy

Intrinsic tyrosine fluorescence was used to measure C‐lobe Ca^2+^ binding affinity. CaM (6 μM) in 50 mM HEPES, 100 mM KCl, 1 mM MgCl_2_, 0.5 mM EGTA, 0.5 mM NTA, pH 7.4 was titrated with defined quantities of CaCl_2_ to achieve free Ca^2+^ concentrations ([Ca^2+^]_free_) ranging from 17 nM to 11 mM across 26 titration points. [Ca^2+^]_free_ were calculated using Maxchelator program.^89^ Experiments were performed on a JASCO FP‐6300 spectrofluorometer (λ_exc_ 277 nm, λ_em_ 300–320 nm) at room temperature. [Ca^2+^]_free_ were validated using Cal520‐FF dye (λ_exc_ 493 nm, λ_em_ 515 nm). Fluorescence emission was normalized and fitted to the Hill equation on GraphPad Prism to calculate the affinity, *K*_d_.

### 5.6 Circular dichroism (CD) spectroscopy

Far‐UV spectra (180–260 nm) for CaM (10 μM) were measured in 2 mM HEPES (pH 7.5) with either 1 mM EGTA, 1 μM CaCl_2_, or 1 mM CaCl_2_ for apo‐ or Ca^2+^‐bound CaM. Measurements were performed in a 0.1 cm path length quartz cell (Hellma Analytics) at 20°C with a scan rate of 100 nm/minute using a JASCO J‐1100 CD spectrometer equipped with a JASCO MCB‐100 mini circulation bath for temperature control. For each sample, three scans were averaged and the buffer baseline was subtracted prior to normalization to mean residue ellipticity (MRE). Secondary structure content was determined by DichroWeb,^90^ using the CDSSTR prediction algorithm, reference data set 7.^91^

Thermal stability of apo/CaM was assessed by monitoring the ellipticity at 222 nm (for α‐helices). Data were collected from 20°C to 80°C in 2°C increments, with a ramp increase rate of 1°C/minute and a 180 s equilibration period between recordings. Data were normalized and fitted to the Boltzmann equation on GraphPad Prism to obtain the melting temperature, *T*_m_.

### 5.7 ^1^H‐ ^15^N heteronuclear single quantum coherence (HSQC) NMR spectroscopy

^1^H‐^15^N HSQC NMR spectra for CaM (100 μM) were measured in 20 mM HEPES, 50 mM NaCl, pH 7.5, 10% D_2_O, supplemented with either 1 mM EGTA (apo/CaM) or 1 mM CaCl_2_ (Ca^2+^/CaM). Experiments were performed at 303 K on a Bruker AVANCE III 700 MHz spectrometer equipped with ^1^H and ^15^N cryoprobes. Data were processed using TopSpin 3.6.1 (Bruker) and analyzed using CcpNmr Analysis Version 2.^92^ For Ca^2+^/CaM, resonance assignments were transferred from those reported by Kainosho and colleagues for CaM‐WT.^93^ Where possible, chemical shift differences for each residue were calculated using the following formula:

$$\Delta \left(H,N\right)=\sqrt{∆H^{2}+{\left(0.15*\mathrm{\Delta Ν}\right)}^{2}}$$

Percentage similarity between Ca^2+^‐bound CaM spectra was determined based on the proportion of overlapping peaks. Peaks were deemed as overlapping if the calculated chemical shift difference was <0.03 ppm, which was approximately the average standard deviation of all chemical shift differences of assignable peaks from the two disease‐associated CaM variants.

### 5.8 Limited proteolysis

Proteolytic stability of CaM was assessed by measuring CaM sensitivity to trypsin degradation. CaM (5 μM) was diluted in 25 mM HEPES, 100 mM NaCl, supplemented with either 10 mM EGTA (for apo/CaM) or 5 mM CaCl_2_ (for Ca^2+^/CaM), pH 7.5. CaM proteins were incubated for 30 minutes at 37°C with 0–10 μg/mL trypsin (apo conditions) or 0–30 μg/mL trypsin (Ca^2+^ conditions). Reactions were rapidly terminated by the addition of Laemmli sample buffer and heating to 95°C for at least 10 minutes. Reaction products were separated by SDS‐PAGE (NuPAGE 4–12% Bis‐Tris, Life Technologies) and stained with Coomassie blue (InstantBlue, Abcam). Gels were imaged on a ChemiDoc XRS+ transilluminator (Biorad) and intact CaM levels was quantified by densitometry using Fiji software.^94^

### 5.9 Isothermal titration calorimetry (ITC)

CaM variants (~50 μM) were titrated against Ca_v_1.2 target peptides at a 10‐fold (Ca_v_1.2‐IQ_1665–1685_) or 20‐fold molar excess (Ca_v_1.2‐NSCaTE_51–67_), in 50 mM HEPES, 100 mM KCl, 2 mM MgCl_2_, pH 7.5, supplemented with 5 mM CaCl_2_ (Ca^2+^‐bound) or 5 mM EGTA (Ca^2+^‐free). For the pre‐association experiments, CaM variants (100 μM) were mixed with an equimolar ratio of Ca_v_1.2‐IQ_1665–1685_ (100 μM) and titrated against 5‐ to 10‐fold molar excess of Ca_v_1.2‐NSCaTE_51–67_.

Titrations consisted of 20 injections of 2 μL peptides into CaM samples over 4 s, with a 180 s recovery time between injections. Experiments were performed at 25°C with a 800 rpm stirring rate. Heat changes were recorded on an automated PEAQ‐ITC system (Malvern) and analyzed using MicroCal PEAQ‐ITC software (Malvern). Data were fitted to a one‐site binding model to provide estimates of stoichiometry (N), dissociation constant (*K*_d_), enthalpy change (ΔH) and entropy change (ΔS).

### 5.10 Cell culture and transfection

HEK293 cells stably expressing the Ca_v_1.2 subunits *α*_1C_, *β*_2b_, and *α*_2_δ_1_ under a tetracycline‐inducible promoter (HEK293‐Ca_v_1.2) were purchased from B'SYS. Cells were cultured at 37°C/5% CO_2_, in Dulbecco's modified Eagle medium/F12 GlutaMAX (Gibco) supplemented with 10% (v/v) fetal bovine serum and 1% (v/v) penicillin–streptomycin. Selection pressure for Ca_v_1.2 subunits expression was maintained using: 100 μg/mL Hygromycin B, 15 μg/mL Blasticidin, 0.4 μg/mL Puromycin, and 100 μg/mL Zeocin. Expression of Ca_v_1.2 was induced with 2.5 μg/mL tetracycline for 24 h prior to experimental use.

Cells at ~ 40% confluency were transfected with 1 μg of pHIV‐CaM‐IRES‐EGFP constructs using Lipofectamine 2000 (Invitrogen) according to the manufacturer's guidelines. Cells were transfected 24–36 h prior to experimental use.

### 5.11 Patch‐clamp electrophysiology

Conventional whole‐cell configuration voltage‐clamp was used to obtain Ca^2+^ and Ba^2+^ current recordings from HEK293‐Ca_v_1.2 cells treated with tetracycline. Currents were recorded using an Axopatch 200B amplifier (Molecular Devices), filtered at 2 kHz and sampled at 10 kHz using a Digidata 1320A interface (Molecular Devices). All recordings were taken at room temperature. Bath solutions consisted of 140 mM NaCl, 5 mM CsCl, 0.33 mM NaH_2_PO_4_, 5 mM glucose, 10 mM HEPES, 1 mM MgCl_2_, pH 7.4 (adjusted with CsOH) containing 2 mM of either CaCl_2_ or BaCl_2_ for the measurement of Ca^2+^ and Ba^2+^ currents, respectively. The internal pipette solution formulation was as follows: 140 mM CsMeSO_4_, 5 mM EGTA, 10 mM HEPES, 1.91 mM CaCl_2_ (for a 100 nM free Ca^2+^ concentration), 1 mM MgCl_2_, 1 mM Na‐ATP, pH to 7.2 (adjusted with CsOH). Patch pipettes were pulled from borosilicate glass (outer diameter 1.5 mm, inner diameter 1.17 mm; Harvard Apparatus) and fire‐polished to give a resistance of 3–5 MΩ once filled with pipette solution.

HEK293‐Ca_v_1.2 cells transiently transfected with CaM were lifted and seeded into a glass‐bottomed bath superfused with Ca^2+^‐containing bath solution. CaM‐expressing cells were identified by EGFP fluorescence (Nikon Eclipse TE200 inverted microscope with epifluorescence attachment). Upon achievement of the whole‐cell configuration, cells were manually compensated for series resistance and capacitance and voltage‐clamped at −60 mV.

Activation of whole‐cell Ca^2+^ currents was investigated using depolarizing voltage steps lasting 300 ms and ranging from −40 to +60 mV. An inter‐sweep interval of 2 s was utilized to allow for channel recovery from inactivation.

For the measurement of steady‐state inactivation, the voltage protocol consisted of a 1 s pre‐pulse at voltages ranging from −60 to +40 mV, followed by a 300 ms test pulse at +10 mV.

Peak current amplitudes were obtained using ClampFit10. Data were normalized to the cell capacitance to give estimates of current density. To obtain the voltages of half‐maximal activation and inactivation (V_50_), data were converted to conductance (G) and fitted to the Boltzmann equation (GraphPad Prism).

Ca^2+^‐dependent inactivation (CDI) of Ca_v_1.2 (f300) was determined by measuring the proportion of current remaining at the end of the 300 ms pulse of the activation protocol, with either Ca^2+^ or Ba^2+^ as the charge carrier. Residual current after 300 ms was divided by peak current to give r300.

$$f300=\frac{r300_{\mathrm{Ba}}-r300_{\mathrm{Ca}}}{r300_{\mathrm{Ba}}}$$

### 5.12 Ethics approval statement

Ethics approval was not required.

### 5.13 Data analysis

Data are presented as mean ± SEM unless stated otherwise. With the exception of NMR spectra, experiments were performed at least in triplicate and analyzed using GraphPad Prism; number of replicates are stated in the relevant figure legends. Significance of data was assessed by one‐way ANOVA or two‐way ANOVA, with Dunnett's post hoc test (*p < 0.05, ^†^ *p* < 0.01, ^‡^ *p* < 0.001, ns > 0.05).

## AUTHOR CONTRIBUTIONS

**Nitika Gupta:** Investigation; formal analysis; writing – original draft; writing – review and editing; conceptualization; methodology. **Ella M. B. Richards:** Investigation; writing – review and editing. **Vanessa S. Morris:** Investigation; writing – review and editing. **Rachael Morris:** Investigation; writing – review and editing. **Kirsty Wadmore:** Investigation; writing – review and editing. **Marie Held:** Investigation; writing – review and editing. **Liam McCormick:** Investigation; writing – review and editing. **Ohm Prakash:** Investigation; writing – review and editing. **Caroline Dart:** Supervision; formal analysis; project administration; conceptualization; investigation; funding acquisition; writing – original draft; writing – review and editing; methodology. **Nordine Helassa:** Conceptualization; methodology; investigation; funding acquisition; project administration; writing – original draft; writing – review and editing; formal analysis; supervision.

## CONFLICT OF INTEREST STATEMENT

The authors declare no conflict of interest.

## Supporting information


Figures S1–S4.

Data S1.

## Data Availability

Data will be made available on request.
