# Supplementary material for: Arrhythmogenic calmodulin variants D131E and Q135P disrupt interaction with the L‐type voltage‐gated Ca2+ channel (Cav1.2) and reduce Ca2+‐dependent inactivation
Source: Acta Physiol (Oxf). 2025 Jan 17;241(2):e14276. doi: 10.1111/apha.14276 (PMC11742489; doi:10.1111/apha.14276)
Supplement: Supplementary file 2 — Data S1. [file APHA-241-e14276-s001.docx]

Figure S1. Arrhythmogenic CaM variants D131E and Q135P show altered Ca**^2^**^+^-CaM structure in low Ca**^2^**^+^ conditions. Average far-UV CD spectra of Ca^2+^-CaM isoforms and relative secondary structure content estimations based on the CDSSTR prediction algorithm (reference data set 7). Data are presented as mean ± SEM (CaM-WT, *n* = 4; D131E, *n* = 4; Q135P, *n* = 4) and differences between groups were determined using a two-way ANOVA with Dunnett's multiple comparisons tests (‡ p < 0.001).

Figure S2. ITC lacks sensitivity to detect binding between apo/CaM and Ca_v_1.2-IQ domain.
Representative ITC titration curves, showing the raw heat changes of the interaction (upper panels) and the integration of the isotherm following baseline correction (lower panels). Experiments were performed at 20°C in Ca^2+^-free conditions (5 mM EGTA).

Figure S3. Apo/CaM does not bind to Ca_v_1.2-NSCaTE domain. Representative ITC titration curves, showing the raw heat changes of the interaction (upper panels) and the integration of the isotherm following baseline correction (lower panels). Experiments were performed at 20°C in Ca^2+^-free conditions (5 mM EGTA).

Figure S4. Arrhythmia-associated CaM variant Q135P pre-bound to Ca_v_1.2-IQ domain, shows decreased binding to Ca_v_1.2-NSCaTE domain. (a) Representative ITC titration curves, showing the raw heat changes of the interaction (upper panels) and the integration of the isotherm following baseline correction (lower panels). (b) Affinity and (c) thermodynamic profile of the interaction of Ca^2+^/CaM:Ca_v_1.2-IQ_1665-1685_ with Ca_v_1.2-NSCaTE_51-67_. Gibbs free energy change (ΔG), enthalpy change (ΔH) and entropy change (-*T*ΔS). Data are presented as mean ± SEM (CaM-WT, *n* = 4; D131E, *n* = 4; Q135P, *n* = 4). Experiments were performed at 20°C in Ca^2+^-saturating conditions (5 mM CaCl_2_). Differences between groups were determined using a one-way ANOVA (for affinity) and two-way ANOVA (for thermodynamics) with Dunnett's multiple comparisons tests (‡ p < 0.001).
